# Acute Otomastoiditis in Children: An Observational Study on the Role of Mastoid Morphology in the Development of Intracranial Complications

**DOI:** 10.3390/jcm14217715

**Published:** 2025-10-30

**Authors:** Camilla Russo, Simone Coluccino, Marco Sarno, Antonia Pascarella, Alida Casale, Antonietta De Lucia, Pietro Spennato, Daniele Cascone, Domenico Cicala, Carmela Russo, Daniele De Brasi, Giuseppe Cinalli, Antonio Della Volpe, Paolo Siani, Eugenio Maria Covelli

**Affiliations:** 1 Neuroradiology Unit, Department of Neuroscience, Santobono-Pausilipon Children’s Hospital, AORN, 80129 Naples, Italy; s.coluccino@santobonopausilipon.it (S.C.); d.cascone@santobonopausilipon.it (D.C.); d.cicala@santobonopausilipon.it (D.C.); c.russo1@santobonopausilipon.it (C.R.); e.covelli@santobonopausilipon.it (E.M.C.); 2 Chronic and Multifactorial Diseases Unit, Department of General and Emergency Pediatrics, Santobono-Pausilipon Children’s Hospital, AORN, 80129 Naples, Italy; m.sarno@santobonopausilipon.it (M.S.); a.pascarella@santobonopausilipon.it (A.P.); a.casale@santobonopausilipon.it (A.C.); p.siani@santobonopausilipon.it (P.S.); 3 Otology and Cochlear Implant Unit, Department of Surgical Specialties, Santobono-Pausilipon Children’s Hospital, AORN, 80129 Naples, Italy; a.delucia@santobonopausilipon.it (A.D.L.); a.dellavolpe@santobonopausilipon.it (A.D.V.); 4 Neurosurgery Unit, Department of Neurosciences, Santobono-Pausilipon Children’s Hospital, AORN, 80129 Naples, Italy; p.spennato@santobonopausilipon.it (P.S.); giuseppe.cinalli@gmail.com (G.C.); 5 Medical Genetics Unit, Department of General and Emergency Pediatrics, Santobono-Pausilipon Children’s Hospital, AORN, 80129 Naples, Italy; d.debrasi@santobonopausilipon.it

**Keywords:** acute otomastoiditis, mastoid, sigmoid sinus, children, intracranial complication, computed tomography, magnetic resonance imaging

## Abstract

**Background**: Acute otomastoiditis (AOM) may occasionally progress to severe intracranial complications in children. While immunological and microbiological factors have been studied, the role of temporal bone anatomical variants remains less well-defined. The aim of this study is to investigate the prevalence of anatomical variants in pediatric patients with acute complicated otomastoiditis (ACOM) compared to those with uncomplicated ones (AUOM) and healthy controls (HC) and assess their potential association with intracranial complication patterns. **Methods**: This retrospective, single-center study reviewed clinical and neuroradiological data of patients aged 0–16 years admitted for AOM between 2018 and 2025. ACOM patients were compared to AUOM and HC groups (the latter undergoing neuroimaging for minor head trauma). Two experienced neuroradiologists evaluated imaging to identify anatomical variants involving the following: (1) sigmoid sinus and emissary veins; (2) tegmen tympani; and (3) mastoid pneumatization. Statistical analyses assessed prevalence differences across groups. **Results**: Among 282 AOM patients, 58 had intracranial complications. Anatomical variants were significantly more frequent in ACOM patients versus both AUOM and HC (*p* < 0.01). In this subgroup, vascular anatomical variants were notably associated with vascular or combined (vascular and infectious) complications; tegmen tympani and mastoid pneumatization variants showed no significant subgroup associations. **Conclusion**: Anatomical variants, particularly vascular anomalies of the sigmoid sinus and emissary veins, appear to increase pediatric AOM patients’ susceptibility to intracranial complications. Recognition of these configurations through early neuroimaging could aid risk stratification and improve diagnostic and therapeutic strategies.

## 1. Introduction

**Figure 1:**
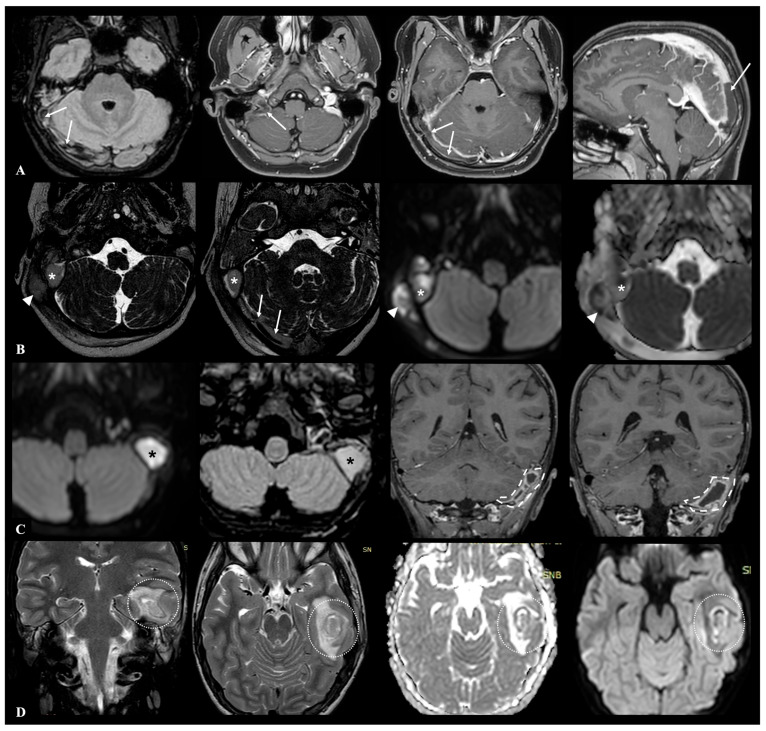
MRI examples of the major possible otogenic intracranial complications in pediatric AOM patients: row (**A**) thrombosis of the transverse-sigmoid-jugular venous complex extending to superior sagittal sinus (white arrows) in a 13-year-old male patient with right-sided ACOM; row (**B**) right-sided ACOM in a 7-year-old female patient, with both extracranial extension (retromastoid abscess located beneath the proximal insertion of the sternocleidomastoid muscle—white arrowheads) and intracranial extension (epidural empyema of the posterior cranial fossa—white asterisks—associated with severe flow reduction in the transverse sinus—white arrows—and meningeal thickening); row (**C**) left-sided posterior cranial fossa subdural empyema (white dashed lines) in a 5-year-old male patient with large otomastoid abscess (black asterisks) and meningeal thickening; row (**D**) left middle cranial fossa brain abscess (white dotted circles) involving the temporal lobe secondary to ACOM in a 4-year-old female patient.

Acute otomastoiditis (AOM) is an infectious condition involving both the middle ear and the mastoid air cells, and it represents the most frequent secondary site of involvement in acute otitis media [1,2]. While most cases resolve without sequelae following appropriate antibiotic therapy, a small proportion may progress to complicated forms with extension of the infection to adjacent structures. These complications, which are broadly categorized as intracranial and extracranial, are associated with significantly increased morbidity, and require prompt recognition and management to prevent serious or permanent outcomes [3]. Intracranial complications, though relatively rare—with an estimated incidence ranging from 0.2% to 2%—are particularly severe and potentially life-threatening [4]. These include thrombosis of the transverse-sigmoid-jugular venous complex, meningitis, epidural empyema, subdural empyema, brain abscess, and in some cases secondary hydrocephalus [5]. An example of possible intracranial complications during AOM is shown in Figure 1.

AOM occurs with significantly greater frequency in children, in whom early identification of individual susceptibility to developing complications is particularly important [1]. Several predisposing factors have been identified that increase the risk of developing intracranial complications in pediatric patients with AOM. One of the most critical factors is the immaturity of the immune system, particularly in younger children whose innate and adaptive immune responses may not yet be fully developed; this can limit their ability to effectively clear bacterial pathogens from the middle ear and surrounding structures. Additionally, both congenital and acquired immunodeficiencies—such as selective IgA deficiency, common variable immunodeficiency, or immunosuppression due to chronic illness or medications—further compromise the host’s defense mechanisms, allowing infections to spread beyond the middle ear. The presence of prothrombotic factors, whether inherited or acquired, may also contribute by promoting the formation of dural venous sinus thrombosis. Moreover, colonization of the nasopharynx or middle ear by particularly virulent or antibiotic-resistant organisms—such as Streptococcus pneumoniae, Haemophilus influenzae, or Pseudomonas aeruginosa—has been linked to more aggressive infections and a higher likelihood of complications. Concurrent viral or bacterial infections can impair muco-ciliary clearance and damage local tissue barriers, facilitating bacterial invasion into the intracranial space. Finally, failure to respond to initial empiric antimicrobial therapy may indicate either resistance or inappropriate coverage, both of which can permit unchecked infection progression. Collectively, these factors highlight the importance of early recognition, appropriate treatment, and careful monitoring of pediatric AOM cases to prevent potentially life-threatening intracranial complications [4,6,7,8,9,10].

However, the role of anatomical factors remains less clearly defined. Indeed, anatomical normal variants, though typically asymptomatic, may in some cases predispose individuals to infections or facilitate the spread of infectious agents. Variations in structural anatomy can create niches or pathways that alter local physiology, reduce immune surveillance, or hinder drainage, thereby increasing susceptibility to infection. For instance, a persistent craniopharyngeal canal—an uncommon but benign variant—can serve as a conduit for nasopharyngeal pathogens to ascend into the intracranial space, potentially leading to recurrent meningitis; similarly, anatomical deviations in the paranasal sinuses, such as concha bullosa or septal deviation, may impair normal muco-ciliary clearance, predisposing individuals to chronic or recurrent sinusitis. While these variants are not inherently pathological, their presence may tip the balance toward disease under certain conditions, especially when combined with other risk factors. Understanding the role of anatomical variation is therefore crucial in explaining individual differences in infection susceptibility and recurrence. Similarly, certain morphological variations in the temporal bone and mastoid region may represent potential predisposing conditions that facilitate the intracranial spread of infection from the otomastoid area. Normal anatomical variants of the mastoid bone [11,12,13,14] that may be worthy of consideration in the evaluation of patients with acute otomastoiditis complications encompass the following:

1. Anatomical variants of the sigmoid sinus and emissary veins [15,16]: variations in the sinus’s depth, course, or position—such as high riding jugular bulb where the roof of the jugular bulb extends more superiorly in the petrous temporal bone than is conventional, or dehiscent jugular bulb where the bulb protrude into the middle ear cavity (from type 3 to type 5 according to Manjila et al. classification of jugular bulb location [17])—can increase the risk of venous thrombosis or facilitate hematogenous spread of infection to intracranial sites;
2. Thickness and position of tegmen tympani [18]: a particularly thin or dehiscent tegmen can increase vulnerability to intracranial extension of infection and complicate surgical access to the middle ear and mastoid regions;
3. Degree of mastoid pneumatization: a hyperpneumatic mastoid (grade III and IV according to Han et al. [19]), characterized by extensive air cell development, may offer less resistance to the dissemination of infection; similarily, accessory mastoid air cells, such as retroauricular or perilabyrinthine cells, can potentially represent anatomical variants that may facilitate the spread of infection to adjacent intracranial or extracranial structures due to their atypical location and potential communication with critical areas.
4. Schematic operational definition of anatomical variants is summarized in Table 1.

With this background, the aim of the present study is to investigate the prevalence of clinically significant anatomical variants in a cohort of pediatric patients hospitalized for acute complicated otomastoiditis with intracranial involvement (ACOM). Specifically, this cohort will be stacked up against two distinct comparison groups to better understand potential anatomical predispositions to severe disease. The first comparison group consists of pediatric patients admitted for acute uncomplicated otomastoiditis (AUOM), representing cases with similar infectious etiology but without intracranial extension. The second group includes healthy controls (HC), selected among children with no history of middle ear or mastoid disease, serving as a baseline reference population.

## 2. Materials and Methods

This study is a retrospective, single-center analysis conducted at Santobono-Pausilipon Children’s Hospital in Naples; Santobono-Pausilipon Children’s Hospital is a major tertiary referral center, serving as regional and extra-regional hub in Southern Italy for pediatric emergency medicine, high-complexity care, and specialized services. Clinical and neuroradiological data were collected through the review of medical records, contrast-enhanced computed tomography (CT) ± magnetic resonance imaging (MRI) reports, and hospital discharge summaries of patients admitted for AOM (with or without evidence of intracranial complications) between January 2018 and January 2025. Hospital admission criteria for AOM included: presence of severity signs such as high fever, poor general condition, and sepsis signs; severe postauricular swelling or abscess; suspicion of intra- or extracranial complications; failure of outpatient oral antibiotic therapy within 24–48 h; very young age (<2 years) or significant comorbidities. In these cases, as the indication for neuroimaging arose, CT and/or MRI were performed promptly to avoid diagnostic delay and to guide surgical/medical interventions; CT of the temporal bone was typically indicated as the first-line imaging modality in the acute phase, while MRI was indicated when intracranial or intratemporal complications were strongly clinically suspected since admission or during hospitalization. Additionally, a large cohort of HCs, comparable for age and sex, was identified; these were patients who, during the same period, underwent brain CT at the same institution due to minor head trauma and showed no evidence of associated osseous or soft tissue injury.

**Figure 2:**
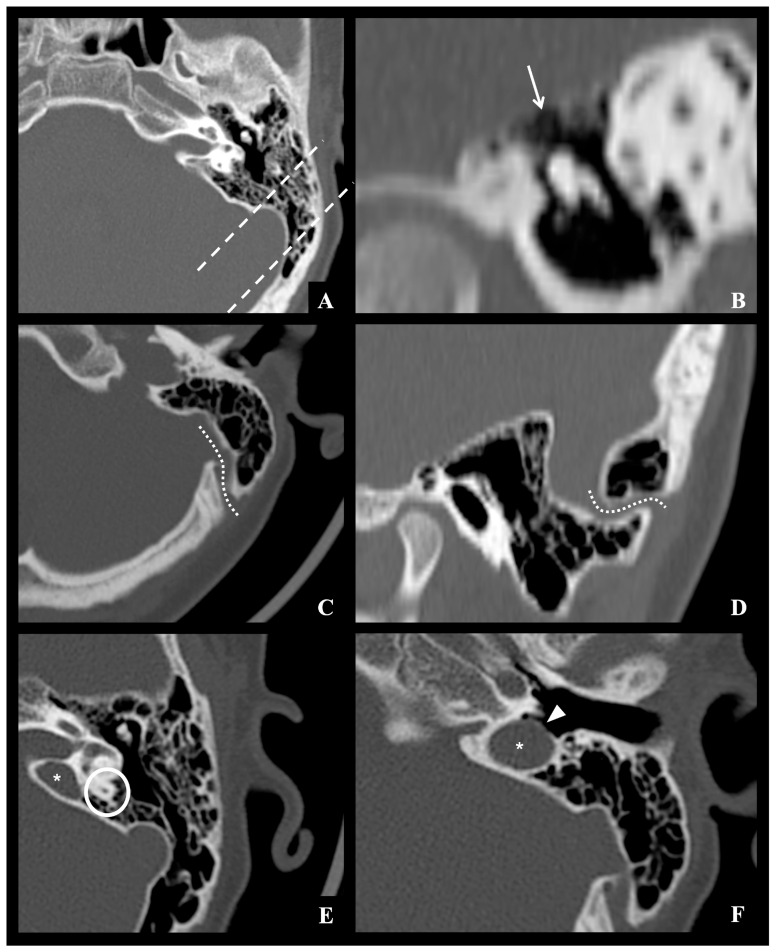
Example of normal mastoid anatomical variants observed on the left side in a same 11-year-old male control patient undergone brain CT examination due to traffic injury: (**A**) grade IV mastoid hyperpneumatization according to Han et al., with air cells extending beyond the sigmoid sinus posteriorly (dashed lines); (**B**) thinned tegmen tympani (white arrow); (**C**,**D**) accessory mastoid emissary vein enlargement (dotted lines); (**E**,**F**) high riding jugular bulb type 4 according to Manjila et al. classification (star), located above the inferior margin of the posterior semicircular canal (white circle), with dehiscent sigmoid plate protruding into middle ear (white arrowhead).

All neuroradiological images were re-evaluated in consensus by two experienced neuroradiologists to identify normal anatomical variants potentially considered predisposing or facilitating the intracranial spread of infection from the otomastoid region. Specifically, these factors were classified as follows: (1) vascular variants involving course, or position of the sigmoid–jugular sinus complex and/or the presence of aberrant mastoid emissary veins (defined as a transosseous connection between the sigmoid dural venous sinus and the suboccipital venous plexus); (2) variants in the thickness of the tegmen tympani; and (3) pneumatization variants, including hyperpneumatization of mastoid air cell compartments and/or the presence of accessory air cells in retroauricular or perilabyrinthine locations. Cases in which inflammatory bone destruction obscured or mimicked normal anatomical variants were carefully reviewed in consensus by two experienced neuroradiologists, and when diagnostic uncertainty persisted, they were excluded from morphometric analysis. An example of the considered anatomical variants is shown in Figure 2.

Inclusion criteria for this study were carefully defined to ensure a homogeneous pediatric population and the reliability of imaging assessments. Eligible participants were required to be between 0 and 16 years of age at the time of hospital admission; only patients who had undergone appropriate imaging studies—CT and, when available, MRI—at the Neuroradiology Unit of the same institution were considered for inclusion, to maintain consistency in imaging protocols and interpretation. Conversely, exclusion criteria included patients aged 16 years or older, as well as those with a clinical history of trauma or previous surgical procedures involving the temporal bone or mastoid region, which could alter anatomical landmarks. Additionally, participants with evidence of congenital malformations that could distort the typical structure of the middle ear, mastoid, or adjacent intracranial spaces were also excluded, as such anomalies could confound the identification of normal anatomical variants relevant to disease progression. The presence of any prosthetic materials or medical devices in the cranial or temporal region that could degrade imaging quality or obscure anatomical structures was also a basis for exclusion. Subjects with known recurrent or previously complicated otomastoiditis were also excluded, as were those lacking suitable neuroimaging studies for radiological review.

All statistical analyses were performed using XLSTAT software, version 2019.2 (Addinsoft, Paris, France). The comparability of AOM patients and HC group in terms of age and sex was preliminarily assessed using the two sample Mann–Whitney U-test and Chi-squared test, respectively. To assess whether the prevalence of anatomical variants differed significantly among the three groups, contingency tables were constructed for each variant and Chi-squared test of independence was then performed; pairwise comparisons between groups were then conducted when overall group differences were statistically significant, with Bonferroni correction applied to account for multiple testing.

The study received formal approval from the local ethics committee “Comitato Etico Territoriale Campania 1” (protocol no. 0024361, dated 19 November 2024). All procedures involving human participants in this study were conducted in full accordance with the ethical standards set forth in the 1964 Declaration of Helsinki and its subsequent amendments, as well as with any applicable national and institutional ethical guidelines, to ensure the safety, rights, dignity, and well-being of all individuals involved in biomedical research. Prior to participation, informed consent was obtained from all patients and/or their legal guardians, as appropriate for a pediatric population; this consent covered both the clinical management and the diagnostic imaging procedures that formed part of the standard care pathway. The informed consent process included a clear explanation of the procedures, potential risks and benefits, the voluntary nature of participation, and the right to withdraw consent at any time without affecting the quality of care received; efforts were made to ensure that information was communicated in an age-appropriate and understandable manner, and that legal guardians were given sufficient opportunity to ask questions and make informed decisions.

## 3. Results

After applying the inclusion and exclusion criteria to the subjects identified through the review of medical records, a total of 282 patients diagnosed with AOM were included in the study (158 males and 124 females; mean age: 4.8 years ± SD 3.2 years; age range: 0.2–14.8 years). These were subdivided into 58 patients with ACOM, accounting for approximately 21% of the total (32 males, 26 females; mean age: 6.1 years ± SD 3.6 years; range: 0.9–14.8 years), and 224 patients with AUOM, representing approximately 79% of the total (126 males, 98 females; mean age: 4.4 years ± SD 3.0 years; range: 0.2–14.8 years). In the ACOM group, the consensus review of contrast-enhanced CT and/or MRI images revealed evidence of phlebitis or thrombophlebitis in 12 of 58 patients (21%), intracranial infectious spread (including meningitis, subdural or epidural empyema, and brain abscess) in 21 of 58 patients (36%) and combined vascular and infectious complications in 25 of 58 patients (43%). For the control group, 282 HCs (179 males, 103 females; mean age: 5.0 years ± SD 3.2 years; range: 0.2–14.6 years) were also identified. Relevant demographic and clinical data for all study participants are summarized in Table 2.

The comparison of age distribution between AOM and HC groups using a two-sample Mann–Whitney U-test with normal approximation showed that the observed difference was not statistically significant (Z = 1.0607, *p* = 0.2888). Similarly, sex distribution comparison between the two groups using the chi-square test revealed no statistically significant difference (χ^2^ = 3.2513, *p* = 0.071365).

When evaluating the prevalence of anatomical variants across the three groups (ACOM, AUOM, and HC), a chi-square test of independence demonstrated a statistically significant difference in distribution (χ^2^ = 9.5843, *p* = 0.0083; significant at *p* < 0.05), suggesting that anatomical variants are not uniformly distributed among groups. Subsequent pairwise Fisher’s exact tests showed that anatomical variants were significantly more prevalent in the ACOM group compared to both the HC group (*p* = 0.0063) and the AUOM group (*p* = 0.0048). No significant difference was observed between the AUOM and HC groups (*p* = 0.8348). After applying Bonferroni correction for multiple comparisons (adjusted alpha = 0.017), the differences between ACOM and both AUOM and HC remained statistically significant.

Given these findings, the ACOM group was further subdivided into three mutually exclusive subgroups based on the type of intracranial complication identified through imaging: (1) vascular (phlebitis or thrombophlebitis), (2) infectious (meningitis, subdural/epidural empyema, and/or brain abscess), and (3) combined (simultaneous vascular and infectious complications). The distribution of specific anatomical variants—including vascular anomalies of the sigmoid sinus or emissary veins, thin or dehiscent tegmen tympani, and mastoid hyperpneumatization—was then compared across these subgroups using chi-square tests. A significant difference was found in the prevalence of vascular anatomical variants across the three subgroups (χ^2^ = 8.2004, *p* = 0.0166; significant at *p* < 0.05); the prevalence of vascular variants across the three groups was as follows: 17/58 jugular bulb protrusion, 2/58 jugular bulb dehiscence, and 2/58 unusually enlarged emissary vein for ACOM group; 16/224 jugular bulb protrusion, 2/224 jugular bulb dehiscence, and 3/224 unusually enlarged emissary vein for AUOM group; 37/282 jugular bulb protrusion, 11/282 jugular bulb dehiscence and 5/282 unusually enlarged emissary vein for HC group.

Conversely, no significant difference was observed in relation to tegmen tympani variants (χ^2^ = 0.6501, *p* = 0.7225) or mastoid hyperpneumatization variants (χ^2^ = 5.9748, *p* = 0.0504). Pairwise Fisher’s exact tests revealed that vascular anatomical variants were significantly more prevalent in patients with vascular complications alone (*p* = 0.0152) and in those with combined vascular and infectious complications (*p* = 0.0194) compared to patients with infectious complications alone. No significant difference was noted between the vascular-only and combined complication subgroups (*p* = 1). After Bonferroni correction for multiple comparisons (adjusted alpha = 0.017), only the comparison between the vascular-only and infectious-only groups remained statistically significant.

All results of the comparative analyses are comprehensively summarized in Table 3; this table provides a clear overview of the main findings, reporting *p*-values and group-wise differences. For full transparency and reproducibility of the statistical analyses, complete contingency tables used for the computation of all statistical tests are also provided in the Supplementary Materials section.

## 4. Discussion

The presented results demonstrated a significantly higher prevalence of normal anatomical variants in pediatric patients with ACOM, particularly those involving vascular structures; this increased frequency was notably higher when compared to both children with AUOM and HCs, suggesting a potential predisposing role of these variants in the development of intracranial complications. Among the complicated cases, vascular anatomical variants (specifically concerning the sigmoid sinus) were especially associated with higher incidence of AOM intracranial complications (with particular reference to phlebitis and thrombophlebitis, both isolated and associated with other intracranial infectious complications such as meningitis, empyema, or abscess formation). Our findings provide evidence that certain morphological “leave me alone” features may significantly contribute to the pathophysiology of infectious-vascular dissemination in the setting of pediatric AOM, thus impacting on the onset of intracranial (potentially life-threatening) complications; these data suggest that such variants may not merely be incidental but rather may act as susceptibility or facilitating factors in the progression of localized otomastoid infections toward serious intracranial involvement, and they should therefore warrant specific consideration during the initial diagnostic assessment of AOM patients presenting for medical evaluation. The evidence gathered in this study also contributes to expanding and reinforcing current knowledge regarding otogenic lateral sinus thrombosis [20], a rare but serious intracranial complication of AOM primarily resulting from the spread of infection to the sigmoid sinus. Anatomically, the sigmoid sinus lies in close proximity to the mastoid air cells, making it particularly vulnerable to contiguous inflammatory processes. These findings suggest how infections can extend through bony dehiscence or via emissary veins that connect extracranial venous systems to the intracranial dural sinuses, thus facilitating retrograde bacterial spread and predisposing to lateral sinus thrombosis especially in pediatric populations with incompletely ossified bone structures. This emphasizes the anatomical vulnerability of the sigmoid sinus and its emissary pathways as critical conduits for septic thrombophlebitis and venous sinus thrombosis in the context of AOM [21,22,23,24].

Conversely, although differences in tegmen tympani thickness/continuity and mastoid hyperpneumatization were more frequently observed in ACOM patients, these did not reach statistical significance within the subgroups of complications; this may reflect either a less direct role of these variants in promoting complications or the influence of other factors such as host immunity, pathogen virulence, or treatment delay. Nonetheless, it was hypothesized a possible impact of mastoid pneumatization and temporal bone morphology on the position of sigmoid sinus and temporal bone-related vessels during skull base development, thus potentially indirectly influencing the higher risk for vascular complications of AOM [25]; therefore the interplay between pneumatized and vascular structures in this region can be more convoluted than expected, and such anatomical complexity may represent an additional challenge in interpreting the potential role of anatomical findings in influencing the spreading of infectious disorders of the temporal bone. It can somehow be assumed that these variants may still pose a risk for intracranial disease extension especially in very young children where this bony barrier is developmentally immature (the evaluation of which should be addressed in future studies involving larger and more specific patient subgroups, stratified according to defined clinical and demographic criteria such as age).

Moreover, these findings also support the paramount importance of neuroimaging techniques, particularly contrast-enhanced CT and MRI, in the diagnostic workup of patients hospitalized for AOM, especially in the early identification of intracranial complications. While CT remains the first-line imaging modality due to its wide availability and ability to delineate bony erosion and mastoid involvement, MRI offers superior soft tissue contrast and is more sensitive in detecting intracranial extension of infection, such as meningitis, abscesses, or venous sinus thrombosis [26]. Previous Authors have emphasized the need for early imaging, even in patients without overt neurological signs, as complications can often be clinically silent or nonspecific in the early stages. According to these recent studies [27,28], clinical symptoms alone may be insufficient to predict intracranial involvement in pediatric AOM and intracranial complications may be present in a subset of patients even despite subtle or absent neurological findings, underscoring the utility of early CT/MRI in selected high-risk cases. More recent findings [11] further support the integration of CT and MRI in the AOM setting to enable timely therapeutic decisions and improve outcomes, also confirming how prompt imaging in case of persistent fever, mastoid swelling, or atypical clinical progression facilitates earlier diagnosis of complications and successfully guides the choice between surgical versus conservative treatment strategies [29,30]. Consequently, such current evidence suggesting a lower threshold for initiating neuroimaging in children with clinical or laboratory indicators of severe otomastoid infection or lack of response to empiric therapy may also support the concurrent identification of susceptibility anatomical factors that may impact on specific intracranial complications. This may simultaneously serve as a valuable diagnostic element for stratifying patients who could benefit from targeted preventive therapies (i.e., anticoagulation), as opposed to those at lower risk [9,10,21,24,31]; in this light, a tailored, patient-specific approach should guide both the timing and selection of therapeutic interventions, taking into account the individual’s clinical presentation, risk profile, underlying anatomical or physiological factors, and response to initial treatment [32]. However, further studies involving larger cohorts are warranted to clarify this controversial issue and support evidence-based clinical decision-making.

Finally, main strength of this study lies in its structured imaging review performed in consensus by two experienced neuroradiologists. Study solidity is also supported by the inclusion of two distinct comparison groups: one composed of pediatric patients with AUOM and another consisting of HCs with no history of middle ear or mastoid disease; this dual comparison design allows for a more nuanced understanding of the potential independent role of anatomical variants in determining disease severity. By contrasting complicated cases not only with milder infectious presentations but also with a baseline healthy population, the study enhances its ability to identify specific anatomical predispositions that may contribute to the progression from uncomplicated to complicated disease. However, certain limitations must be acknowledged. The retrospective design inherently limits causal inference. The identification and hypothetical physiological involvement of anatomical variants was based solely on imaging without pathological confirmation, although CT and MRI are widely accepted as reliable diagnostic tools. In some cases, inflammatory bone destruction may have partially obscured or mimicked normal anatomical variants, potentially limiting the precision of morphological assessment despite expert consensus review. Another potential limitation of this study is the unbalanced distribution of age sub-groups within the pediatric cohort, which may have limited the ability to assess the influence of developmental differences in temporal bone anatomy—i.e., between “younger” and “older” children—on the observed findings. Moreover, although statistical corrections were applied to control for multiple comparisons, the relatively small number of ACOM cases in some subgroups may have limited the statistical power to detect other meaningful associations. Finally, a possible limitation of this study is the exclusion of mastoid hypopneumatization variants, given the current lack of standardized pediatric criteria for their definition and the high interindividual variability in mastoid pneumatization with age [33]; thus, to ensure methodological rigor and reproducibility, this variable was intentionally excluded from the present analysis, and future studies integrating age-adjusted volumetric assessment protocols are warranted to define the potential contribution of mastoid hypopneumatization in disease susceptibility and intracranial AOM complication risk.

As final considerations, in this study the role for mastoid anatomical variants in AOM intracranial complications has been investigated as an independent risk factor; however, as such complications are frequently multifactorial, further studies that condense the contribution of different predisposing or susceptibility factors (i.e., health condition, immune status, concomitant therapies, vaccines, and so on) are still required. In addition, despite not included in present study, recurrent or previously complicated cases of AOM (which often represent the most clinically challenging scenarios) may be similarly influenced by underlying anatomical mastoid variants that predispose to impaired drainage, altered ventilation, or proximity to critical intracranial structures. Although this hypothesis aligns with the observed complexity and recurrence of certain cases, the present study was not designed to address this aspect; therefore, the potential contribution of mastoid anatomical variants to the intracranial extension of recurrent otomastoiditis warrants dedicated investigation in future studies.

## 5. Conclusions

This study highlights the critical role of potentially predisposing normal anatomical variants in modulating the spread of infection and contributing to the development of intracranial complications in pediatric cases of AOM. In particular, morphological variants involving the venous structures of the temporal bone—most notably, the sigmoid sinus and the mastoid emissary veins—appear to play a significant role in facilitating the extension of infection beyond the middle ear and mastoid air cells. These anatomical structures, which naturally serve as conduits for venous drainage, may under certain configurations become vulnerable pathways for retrograde spread of septic thrombophlebitis. Variants such as a laterally displaced sigmoid sinus, a high-riding jugular bulb, enlarged mastoid emissary veins, or dehiscence of the sinus wall may increase the risk of intracranial extension of mastoid infections. In the context of AOM, these features may create conditions favorable for the hematogenous translocation of infectious agents into the dural venous sinuses, cranial cavity, or central nervous system. As such, they represent potential independent anatomical risk factors for serious complications including otogenic lateral sinus thrombosis and septic thrombophlebitis. The findings of this study underscore the importance of identifying such variants during early diagnostic work-up in children presenting with AOM; recognition of these risk-enhancing anatomical features on imaging can support clinical decision-making, prompt more aggressive diagnostic or therapeutic interventions, and contribute to timely referrals to specialized care. Early detection and appropriate management may ultimately help reduce the risk of irreversible neurological damage and improve overall outcomes in this vulnerable population. Despite these findings highlight the importance of careful imaging assessment in children presenting with AOM, they should nonetheless be interpreted in light of the study’s limitations; future prospective, multicentric, and age-stratified studies are needed to validate these associations and further elucidate the mechanistic role of temporal bone variants in the pathophysiology of otogenic complications.

## Figures and Tables

**Table 1:** Operational definition of anatomical variants relevant for the study.

**1.**	**Vascular variants**		
	*(a) Vascular variants involving course, or position of the sigmoid–jugular sinus complex (according to Manjila* et al. *classification)*		
		Type 1: no jugular bulb present.	
		Type 2: jugular bulb protrusion below the inferior margin of the posterior semicircular canal.	
			2a: Without middle ear dehiscence.
			2b: With dehiscence into the middle ear.
		Type 3: jugular bulb between inferior margin of posterior semicircular canal and inferior margin of internal auditory canal.	
			3a: Without middle ear dehiscence.
			3b: With dehiscence into the middle ear.
		Type 4: jugular bulb located above the inferior margin of the internal auditory canal.	
			4a: Without dehiscence into the internal auditory canal.
			4b: With dehiscence into the internal auditory canal.
		Type 5: combination of dehiscences.	
	*(b) Vascular variants involving the presence of aberrant mastoid emissary veins*		
		Unusually prominent or enlarged mastoid emissary vein.	
**2.**	**Tegmen tympani variants**		
		Normal tegmen tympani: typically measured around 1 to 2 mm.	
		Papyraceous/Thin Tegmen: very thin or “paper-like” tegmen tympani without a full dehiscence.	
		Tegmen Dehiscence: complete absence of bone in a portion of the tegmen.	
**3.**	**Mastoid pneumatization variants (according to Han et al. classification)**		
		Grade I: confined to anteromedial region of an arbitrary line passing through the most anterior point of the sigmoid sinus.	
		Grade II: between two arbitrary lines passing through the most anterior and the most lateral aspect of the sigmoid sinus.	
		Grade III: between two arbitrary lines passing through the most lateral and the most posterior aspect of the sigmoid sinus.	
		Grade IV: extended beyond an arbitrary line passing through the most posterior aspect of the sigmoid sinus.	

**Table 2:** Relevant demographic and clinical data of all study participants.

		AOM (Overall)	ACOM	AUOM	HC
**Demographics**		282	58	224	282
	M	158	32	126	179
	F	124	26	98	103
	Age (mean)	4.8	6.1	4.4	5.0
	SD	3.2	3.6	3.0	3.2
	Min	0.2	0.9	0.2	0.2
	Max	14.8	14.8	14.8	14.6
**Extracranial complications**		39 (14%)	17 (29%)	22 (10%)	-
**Intracranial complications**		59 (21%)	58 (100%)	-	-
	Vascular (phlebitis or thrombophlebitis)	11 (4%)	12 (21%)	-	-
	Infectious (meningitis, empyema, abscess)	20 (7%)	21 (36%)	-	-
	Combined	25 (9%)	25 (43%)	-	-

*Legend: M = males; F = females; SD = standard deviation; AOM = acute otomastoiditis; ACOM = acute complicated otomastoiditis (with intracranial complications); AUOM = acute uncomplicated otomastoiditis; HC = healthy controls.*

**Table 3:** Results of all the statistical analyses performed on the groups and subgroups under examination; a *p*-value < 0.05 is considered statistically significant; statistically significant results are highlighted in italics; * *not* significant after Bonferroni correction.

**Statistics**			***p*-Value**
Overall prevalence of anatomical variants			
Chi-squared test of independence among the three groups (ACOM vs. AUOM vs. HC)			***0.0083***
	→ Pairwise Fisher’s Exact test between groups		-
		ACOM vs. HC	***0.0063***
		ACOM vs. AUOM	***0.0048***
		AUOM vs. HC	0.8348
Individual prevalence of anatomical variants			
Chi-squared test of independence among ACOM subgroups (vascular vs. infectious vs. combined)			
		vascular anatomical variants	***0.0166***
		tegmen tympani variants	0.7225
		mastoid hyperpneumatization variants	0.0504
	→ Pairwise Fisher’s Exact test of vascular anatomical variants between ACOM subgroups		-
		vascular vs. infectious	***0.0152***
		vascular vs. combined	1
		infectious vs. combined	0.0194 *

*Legend: ACOM = acute complicated otomastoiditis (with intracranial complications); AUOM = acute uncomplicated otomastoiditis; HC = healthy controls.*

## Data Availability

Data available on request due to restrictions (e.g., privacy and ethical reasons—minor patients).

## Supplementary Materials

The following supporting information can be downloaded at: https://www.mdpi.com/article/10.3390/jcm14217715/s1, File S1: Complete contingency tables of all the statistical analyses performed on the groups and subgroups under examination.

## Abbreviations

ACOM	acute complicated otomastoiditis (with intracranial involvement)
AOM	acute otomastoiditis
AUOM	acute uncomplicated otomastoiditis
CT	computed tomography
HC	healthy control
MRI	magnetic resonance imaging
SD	standard deviation
