# Supplementary material for: Acute Otomastoiditis in Children: An Observational Study on the Role of Mastoid Morphology in the Development of Intracranial Complications
Source: J Clin Med. 2025 Oct 30;14(21):7715. doi: 10.3390/jcm14217715 (PMC12608297; doi:10.3390/jcm14217715)
Supplement: Supplementary file 1 [file jcm-14-07715-s001.zip › jcm-3834879-supplementary.pdf]

Acute Otomastoiditis in Children: An Observational Study on the Role of Mastoid Morphology in the Development of Intracranial Complications.

Supplementary Materials File S1. Complete contingency tables of all the statistical analyses performed on the groups and subgroups under examination.

Chi-squared test of independence among the three groups (ACOM vs AUOM vs HC)

	anatomical variants (overall) +	anatomical variants (overall) -	***Row Totals***
ACOM	25 (15.22) [6.28]	33 (42.78) [2.24]	58
AUOM	53 (58.78) [0.57]	171 (165.22) [0.20]	224
HC	70 (74.00) [0.22]	212 (208.00) [0.08]	282

The chi-square statistic is 9.5843. The *p*-value is .008295. The result is significant at $p < .05$

Pairwise Fisher's Exact test between groups

1) ACOM vs HC

	anatomical variants (overall) +	anatomical variants (overall) -	***Marginal Row Totals***
ACOM	25	33	58
HC	70	212	282

The Fisher exact test statistic value is 0.0063. The result is significant at $p < .05$.

2) ACOM vs AUOM

	anatomical variants (overall) +	anatomical variants (overall) -	***Marginal Row Totals***
ACOM	25	33	58
AUOM	53	171	224

The Fisher exact test statistic value is 0.0048. The result is significant at $p < .05$.

3) AUOM vs HC

	anatomical variants (overall) +	anatomical variants (overall) -	***Marginal Row Totals***
AUOM	53	171	224
HC	70	212	282

The Fisher exact test statistic value is 0.8348. The result is *not* significant at $p < .05$.

Chi-squared test of independence among ACOM subgroups (vascular vs infectious vs combined)

1) vascular anatomical variants

	vascular anatomical variants +	vascular anatomical variants -	***Row Totals***
ACOM with vascular complications	6 (3.93) [1.09]	6 (8.07) [0.53]	12
ACOM with infectious complications	2 (6.88) [3.46]	19 (14.12) [1.69]	21
ACOM with combined complications	11 (8.19) [0.96]	14 (16.81) [0.47]	25

The chi-square statistic is 8.2004. The *p*-value is .016569. The result is significant at $p < .05$.

2) tegmen tympani variants

	tegmen tympani variants +	tegmen tympani variants -	***Row Totals***
ACOM with vascular complications	1 (0.81) [0.04]	11 (11.19) [0.00]	12
ACOM with infectious complications	2 (1.42) [0.23]	19 (19.58) [0.02]	21
ACOM with combined complications	1 (1.76) [0.33]	25 (24.24) [0.02]	26

The chi-square statistic is 0.6501. The *p*-value is .722502. The result is *not* significant at $p < .05$.

3) mastoid hyperpneumatization variants

	mastoid hyperpneumatization variants +	mastoid hyperpneumatization variants -	***Row Totals***
ACOM with vascular complications	1 (2.07) [0.55]	11 (9.93) [0.12]	12
ACOM with infectious complications	7 (3.62) [3.15]	14 (17.38) [0.66]	21
ACOM with combined complications	2 (4.31) [1.24]	23 (20.69) [0.26]	25

The chi-square statistic is 5.9748. The *p*-value is .050418. The result is *not* significant at $p < .05$.

Pairwise Fisher's Exact test of vascular anatomical variants between ACOM subgroups

1) vascular vs infectious

	vascular anatomical variants +	vascular anatomical variants -	***Marginal Row Totals***
ACOM with vascular complications	6	6	12
ACOM with infectious complications	2	19	21

The Fisher exact test statistic value is 0.0152. The result is significant at $p < .05$.

2) vascular vs combined

	vascular anatomical variants +	vascular anatomical variants -	***Marginal Row Totals***
ACOM with vascular complications	6	6	12
ACOM with combined complications	11	14	25

The Fisher exact test statistic value is 1. The result is *not* significant at $p < .05$.

3) infectious vs combined

	vascular anatomical variants +	vascular anatomical variants -	***Marginal Row Totals***
ACOM with infectious complications	2	19	21
ACOM with combined complications	11	14	25

The Fisher exact test statistic value is 0.0194. The result is significant at $p < .05$.
Not significant after Bonferroni correction.

Legend: ACOM=acute complicated otomastoiditis (with intracranial complications); AUOM=acute uncomplicated otomastoiditis; HC=healthy controls.
